# Inhibitory effect of fermented avocado seed extract (*Persea americana* Mill. cv. Hass) on polyphenol oxidase and its application as anti-browning agent in avocado, apple, and banana pulps

**DOI:** 10.1016/j.heliyon.2025.e42588

**Published:** 2025-02-10

**Authors:** Diana Paola Yepes-Betancur, Isabel Cristina Zapata-Vahos, Juan Camilo Henao-Rojas, Jaison Martinez-Saldarriaga, Carlos Julio Márquez-Cardozo, Edith Marleny Cadena-Chamorro

**Affiliations:** a Servicio Nacional de Aprendizaje, SENA, Grupo de Investigación en Innovación y Agroindustria, Centro de la Innovación, la Agroindustria y la Aviación, Cra 48 # 49-62, Rionegro, 054040, Colombia; b Facultad de Ciencias de la Salud, Grupo de Investigación Atención Primaria en Salud, Universidad Católica de Oriente, Rionegro, 054040, Colombia; c Corporación Colombiana de Investigación Agropecuaria–Agrosavia, Centro de Investigación La Selva, Kilómetro 7, Vía a Las Palmas, Vereda Llanogrande, Rionegro, 054048, Colombia; d Grupo de Investigación en Ingeniería Agrícola. Escuela de Biociencias. Universidad Nacional de Colombia, AA 3840, Medellín, Colombia

**Keywords:** Enzymatic browning, Anti-Browning agents, Polyphenolic compounds, Inhibition kinetics, Fermented avocado seed extract, Polyphenoloxidase

## Abstract

Antioxidant components of avocado seed can be used to prevent browning caused by polyphenol oxidase (PPO). This research evaluated the inhibitory effect of avocado seed extract obtained through solid state fermentation on PPO and assessed the anti-browning performance of this extract on avocado, banana, and apple pulp in comparison to ascorbic acid and L-Cysteine. The Km and Vmax kinetic parameters of avocado PPO, using catechol as substrate, were 0.1627 g/L and 0.6577 Abs/min, respectively. The fermented extract completely inhibited the enzyme at 60 % v/v, likewise, the type of competitive inhibition of the fermented extract was determined by the Lineweaver-Burk method. Additionally, fermented extract effectively delayed browning in avocado, and banana at 0.5 % v/v and 1.0 % v/v in apple, the effect is linked to the presence of bioactive compounds as potential inhibitors capable of retarding the darkening. Mathematical modeling of enzyme inhibition was performed and it was found self-inhibition is present in most cases. In conclusion, fermented avocado seed extract is a potential product to protect other foodstuffs from oxidation in the food industry.

## 1 Introduction

Enzymatic browning has a great impact on the quality of some fresh fruits and vegetables, thus limiting their consumption and processing due to the alteration of color, flavor, texture, as well as nutritional value [1]. The browning reaction by Polyphenol Oxidase (PPO) can occur in two ways; monophenol activity, in which a hydroxylation of monophenols to o-diphenols occurs and/or diphenolase activity, where there is an oxidation of o-diphenols to produce o-quinones, followed by polymerization reactions not catalyzed by the enzyme generating melanin pigments [1,2]. The active site of the PPO consists of a pair of copper ions joined by three histidine residues, where the interaction with molecular oxygen and phenolic substrates occurs, thus initiating the enzymatic browning reaction [3].

Plant tissues that are cut, chopped, or bruised suffer a rapid darkening due to cell compartment damage and oxygen penetration, which leads to contact with the PPO-substrate [4]. This factor limits the processing of fruits and vegetables susceptible to enzymatic browning such as avocado [[5], [6], [7], [8]], apple [[9], [10], [11], [12]], plum [13,14] banana [15,16], lettuce [4,17], potato [18], among others.

There are different alternatives to prevent or delay the formation of pigments typical of enzymatic browning, including the addition of inhibitors or compounds that act directly on the PPO, or react with oxygen or phenolic compounds, or with the products generated from enzymatic catalysis. Ascorbic acid and L-Cysteine are widely used as anti-browning agents. Ascorbic acid reduces o-quinones to their original phenols; however, its reducing effect is temporary due to its oxidation when reacting with intermediate pigments, endogenous enzymes, and metals such as copper [19]. L-Cysteine is a sulfur-containing amino acid, that can react directly in the active site of PPO and form stable complexes with copper; nonetheless, it also reacts with o-quinone products to form colorless compounds [19,20].

Several methods have been studied to control the action of the PPO enzyme, from physical methods such as heating [21] and high hydrostatic pressure [22], as well as the addition of chemical compounds such as sulfite sodium, gentisic acid [23], ferulic acid among others [24] or addition of organic acids from natural sources like citric, ascorbic, oxalic, malic and cinnamic acid [25] or polyphenolic compounds [26]. These compounds can be present in extracts of onion, green tea, grape leaf extract, black bean pericarp, among others [[27], [28], [29], [30], [31]]. For example, some authors have reported several components in the avocado seed like: (−)-Epicatechin, quercetin, benzoic acid, caffeic acid, chlorogenic acid, and cinnamic acid, among others [32]. The extract obtained through fermentation with this type of bioactive compound can help maintain their visual appeal and nutritional quality during storage and processing, extend the shelf life of food products by inhibiting the growth of spoilage microorganisms, provide unique tastes and aromas, contribute to the overall sensory experience of the food, enhance their nutritional value and health benefits [33]. Therefore, the objective of this work was to evaluate the inhibition of PPO enzyme using an extract obtained through solid-state fermentation of avocado seed (*Persea americana* Mill. cv. Hass) with *Aspergillus niger.* The inhibition types of representative phenolic inhibitors on PPO were estimated by Lineweaver- Burk double reciprocal plots. Additionally, the anti-browning effects of the fermented extract of avocado seed were valued on three food matrices: avocado, apple, and banana pulp.

## 2 Materials and methods

### 2.1 Plant material

Fruits of avocado (*Persea americana* Mill, cv Hass) in a state of optimum maturity [34] were processed for the extraction of PPO. The enzymatic browning index was evaluated on avocado (*Persea americana Mill*, cv Hass), apple (*Malus domestica*, Royal Gala), and banana (*Cavendish valery*) pulp. The fruits were obtained from a local market located in the municipality of Rionegro, Antioquia, Colombia.

### 2.2 Extraction of the polyphenol oxidase enzyme

The extraction of PPO was performed from avocado pulp previously homogenized in an Ultra-Turrax (IKA® T25) at 3500 rpm for a minute. 8 g of previously homogenized pulp was mixed with 40 mL of 0.01M phosphates buffer (pH: 7.0) and 0.4g of Polyvinylpyrrolidone (Panreac, Germany). This mixture was homogenized in a vortex (Lab Scient® V8) at 1500 rpm for 3 min and centrifuged at 10500 rpm, 0 °C for 10 min in a centrifuge (Hermle Z366K). The obtained supernatant was filtered through 0.45 μm acrodiscs (Sartorius®) to eliminate turbidity in the extract [[21], [35]]. Subsequently, the supernatant was collected and stored at −30 °C for use as crude PPO enzyme for subsequent enzymatic activity determinations.

### 2.3 Enzymatic activity and kinetic parameters

Different concentrations of catechol ([s]) (Alfa Aesar, USA) as a substrate for the enzyme in a range of 0.3 and 4.9 g/L (2.7 a 44.5 mM) were evaluated using phosphate buffer (pH 7.0) [21]. To 100 μL of crude PPO extract (crude extract of avocado pulp) was added the catechol substrate solutions for a total reaction volume of 1160 μL. Readings were taken at 420 nm for 6 min at 20-s intervals in a (Genesys 10S) spectrophotometer using phosphate buffer (pH 7.0) as blank. Enzymatic activity was expressed as the change in absorbance per unit time [21] (Eq. (1)).

(1) $$V=\frac{\Delta Absorbance\left(420nm\right)}{\min\;}*4\;\left(Dilution\;factor\right)$$

The dilution factor (1:4, v/v) was performed with a pH 7.0 phosphate buffer, to the absorbance values of the test solutions were within the linear spectrophotometric range.

Km and Vmax, as kinetic parameters of PPO were obtained through the linearization (Lineweaver & Burk) of the kinetic equation of Michaelis-Menten [21], (Eq. (2)).

(2) $$\frac{1}{v}=\left(\frac{Km}{Vmax}\right)\frac{1}{\left[S\right]}+\frac{1}{Vmax}$$

Where,

The slope of the straight line is Km/Vmax

The intersection in 1/v is 1/Vmax

### 2.4 Fermented extract obtention

In a previous study, a solid-state fermentation of avocado seed (*Persea Americana* Mill. cv. Hass) with *Aspergillus niger* GH1 was carried out [36]. The sample with particle size ≤2.5 mm, humidity of 60 %, and 168 h of fermentation showed the highest ABTS and DPPH radical scavenging capacity, observing an increase of 33 % and 77 %, respectively, in comparison with time zero corresponding to the uninoculated seed (1534.47 mg TE/100 g). For the extraction of phenolic compounds briefly, 4 g of fermented material was dissolved in 15 mL of an ethanol/water solution (56:44, v/v). The mixture was heated in a water bath at 60 °C for 20 min and then centrifuged for 10 min to 1000 g in a Hermle Z366 K centrifuge (Gosheim, Germany). The extracts were filtered by cellulose filters of 0.2 mm and stored at −18 °C until analyzed.

### 2.5 Inhibition of the enzymatic activity of PPO

An extract obtained at 168 h of fermentation in the solid state of avocado seed with *Aspergillus niger* GH1 was evaluated as an inhibitory agent of PPO in concentrations of 10 at 60 % v/v, and the preparation was carried out in phosphate buffer (pH 7.0). The follow-up of the inhibition of the fermented extract was performed through the enzymatic activity of PPO; the total volume of the reaction was 1160 μL, where 833 μL corresponded to the fermented extract for each of the concentrations studied, 100 μL of extract of PPO (crude extract of avocado pulp) and finally 227 μL of catechol 200 mM. The inhibition results of fermented extract were contrasted with ascorbic acid at concentrations between 1.0 and 7.5 % v/v, and L-Cysteine, between 0.5 and 6.0 %v/v.

The inhibition percentage of PPO (%IPPO) was determined by eq. (3) [37].

(3) $$\%IPPO=\frac{E.A.\;control-E.A.\;sample}{E.A.\;control}*100$$

Where, E.A.: enzymatic activity.

### 2.6 Browning index (BI)

Banana, apple, and avocado fruits were peeled, chopped (2 x 2 x 0,3 cm), and immersed for 10 min in solutions at 0.1, 0.5, and 1.0 % (w/v) for each inhibitory agent (fermented extract, ascorbic acid, and L-Cysteine). Subsequently, the chopped fruits were exposed to environmental conditions. During 4 h, chromatic readings were taken in the CIE-L∗a∗b∗ system (L∗, corresponds to luminosity; a∗ colors between green and red; b∗ colors between blue and yellow) in a spectrophotometer (Konica Minolta CR400 - illuminant D65, observer 2°) to determine the Browning Index (BI) according to eqs. (4), (5)) [38]. The results were contrasted with control treatments (distilled water) in the absence of inhibitory agent.

The browning index data was analyzed using a multifactor ANOVA test with a 95 % confidence level in the Statgraphic Centurion XVI.I software.

(4) $$BI=\frac{\left(100*\left(X-0.31\right)\right)}{0,17}$$

(5) $$X=\frac{\left(a+1.75L\right)\;}{\left(5.645L+a-3.012b\right)}$$

Where:

The L index is lightness, a indicates reddish-greenish and b indicates yellowish-bluish.

Also, from the L, a, and b values, colorimetry unified vector kinetics figures were constructed using the free color converter (https://www.nixsensor.com/free-color-converter/).

### 2.7 Mathematical modeling of the change in the browning index (BI)

To adjust the behavior of the variation of the Browning Index with the three treatments experimented, an adjustment was made to a deterministic, non-segregated, and dynamic mathematical model based on the ordinary differential equation proposed by Montoya [39] and adapted for variation of CIELab colorimetric coordinates by Henao-Rojas [40], where the stabilization phase of the BI is represented as a relationship between the maximum Browning Index (BImax) and the registered variation of the Browning Index in a given time (BI), in addition to a self-inhibition factor n, which is interpreted as follows: Where n < 1 browning kinetics is relatively sensitive to self-inhibition and occurs for low BI values, n = 1, logistic equation and n > 1, browning kinetics is relatively resistant to self-inhibition and occurs when BI=BImax (eq. (6)). The above was done in the lower BI value concentrations.

(6) $$\frac{dBI}{dt}=Vmax.BI\left(1-{\left(\frac{BI}{BImax}\right)}^{n}\right)$$

Additionally, a non-linear regression was performed to obtain the kinetic parameters, where the ode45 command was used, which is based on the explicit Runge-Kutta (4), (5) using a Dormand-Prince pair and the ode15s command based in a formula of variable order that works by numerical differentiation formulas [41], using the software Matlab® (MathWorks, USA).

### 2.8 Statistical analyses

Data linearization techniques were applied to obtain the kinetic parameters Km and Vmax, using the Lineweaver-Burk approach based on the Michaelis-Menten equation [21]. Statistical analyses of inhibitory activity and browning index (BI) were performed using multivariate ANOVA tests with a confidence level of 95 % using Statgraphic Centurion XVI.I software, which allowed a rigorous evaluation of the variability and significance of the effects of treatment. The mathematical modeling of the BI was fitted using a dynamic model based on ordinary differential equations [26], with kinetic parameters derived using non-linear regression in Matlab® (MathWorks, USA).

## 3 Results and discussion

### 3.1 Enzymatic activity of PPO and kinetic parameters

**Fig. 1:**
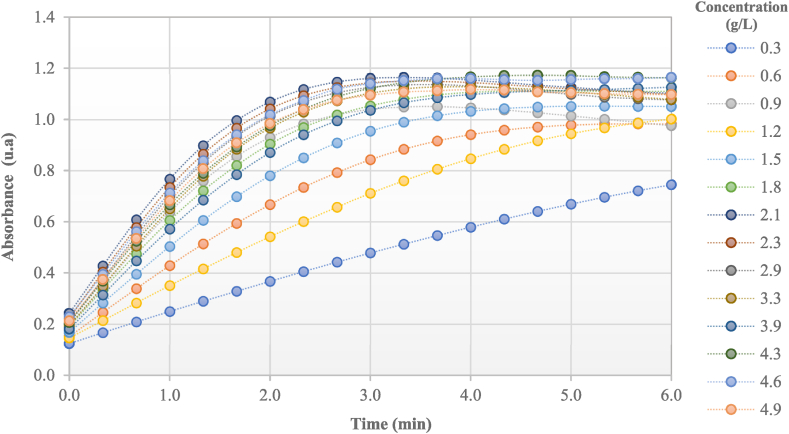
The behavior of the reaction speed of the PPO present in the Hass avocado fruit.

The crude extract of avocado pulp exhibited significant PPO activity in a wide range of concentrations of catechol as a substrate. Enzymatic kinetics reveal a growing trend until 3 min (Fig. 1) with subsequent stabilization. It was observed that not always when starting from a higher concentration of substrate there is a correlation with the absorbance change. There seems to be some ratios of crude extract and substrate that perform better than others. The previous analysis indicates that in the extract there are molecules that exert dual effects on the enzymatic activity acting as accelerators of the enzymatic reaction and as inhibitory agents, which depends on their chemical structure and the reaction environment. Likewise, other compounds have been reported with inhibitory activity against browning such as cinnamic, citric, and malic acids [42,43].

PPO can act as a tyrosinase (EC 1.14.18.1) or as a catechol oxidase (EC 1.10.3.1). Tyrosinase can catalyze two different reaction cycles: the first consists of the ortho-hydroxylation of monophenols to diphenols, followed by the oxidation of the diphenol to o-quinone; the second cycle is the oxidation of an o-diphenol to o-quinone. In contrast, catechol oxidase can only catalyze the oxidation of o-diphenols to o-quinones. Many phenolic compounds have an o-diphenolic group, making them potential substrates for PPO [44]. In other studies, substrate specificity has been characterized for PPO derived from a wide variety of fruits, vegetables, and other plant products, but interestingly, PPO obtained from these foods has only shown catechol oxidase activity [45].

The highest reaction speed of the enzyme was given at 4.3 g/L of catechol (Table 1), at higher substrate concentration a decrease in enzymatic activity occurred, possibly as a consequence of substrate over-saturation or inhibition by product. The reported values of enzymatic activity of PPO change depending on the variety of avocados or the type of fruit evaluated.

**Table 1:** Reaction speeds of PPO at different concentrations of catechol.

Concentration (g/L)	Reaction speed (Abs/min)
0.3	0.414 ± 0.032
0.6	0.555 ± 0.005
0.9	0.570 ± 0.009
1.2	0.570 ± 0.009
1.5	0.591 ± 0.012
1.8	0.616 ± 0.006
2.1	0.615 ± 0.012
2.3	0.618 ± 0.004
2.9	0.620 ± 0.007
3.3	0.623 ± 0.004
3.9	0.626 ± 0.015
4.3	0.646 ± 0.019
4.6	0.624 ± 0.003
4.9	0.603 ± 0.002

**Fig. 2:**
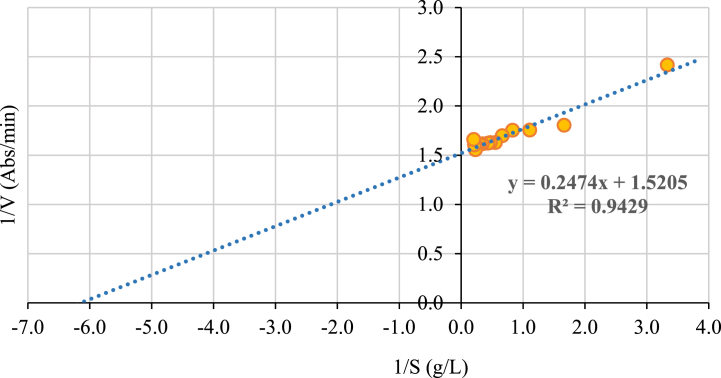
Lineweaver-Burk linearization of PPO present in the crude extract of Hass avocado pulp.

The kinetic parameters determined by the Lineweaver-Burk linearization (Fig. 2.) correspond to a Km of 0.1627 g/L (equivalent to 1.48 mM of substrate) and Vmax of 0.6577 Abs/min, the values are comparable to those reported by Amaya et al. (2008) [46] who evaluated the kinetics of the PPO extracted in Hass Avocado with catechol and DL-DOPA as a substrate. Nonetheless, Kahn, (1976) [26] found that the PPO of Fuerte and Lerman avocado present higher Km values (7.3 and 2.4 mM, respectively) using catechol; and the PPO extracted from the Booth and Julio Millán varieties, Km values of 10.4 and 9.24 mM, respectively [47]. The specific value of Km depends on the variety of the fruit, which indicates that different isoforms of PPO are produced according to the species of the plant, in addition, the value of Km can feature fluctuations according to the method used for the enzyme extraction [21,[42], [48]].

Dopamine, catechin, caffeic acid, and chlorogenic acid are present in the avocado and these are likely the main substrates for PPO in the fruit even though they are present at low concentrations, therefore the enzyme extracted from the fruit exhibits affinity when evaluated with such substrates [21].

### 3.2 Inhibition of the enzymatic activity of PPO

**Fig. 3:**
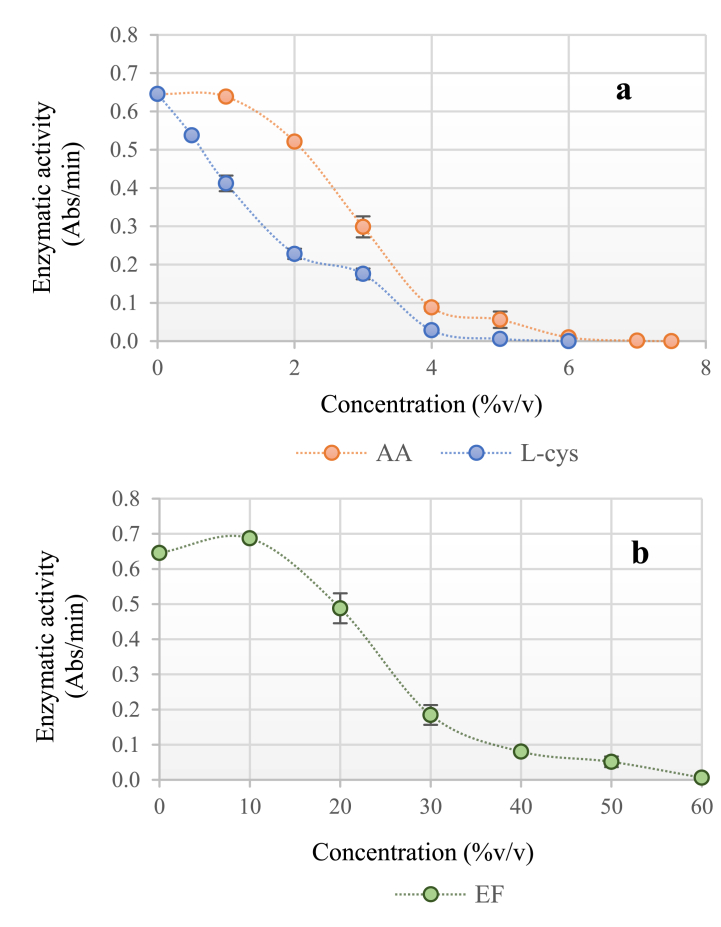
Effect of inhibitors on the enzymatic activity of the PPO. **a.** Ascorbic acid (AA) and L-Cysteine (L-Cys). **b.** Fermented Extract (FE).

The application of the fermented extract on PPO generates an inhibition of 60 % v/v to 99.96 % (Fig. 3b.). Ascorbic acid and L-Cysteine had a complete inhibition at 7.5 % v/v (0.75 mM) and 6.0 % v/v (0.6 mM), respectively (Fig. 3a.). The most effective inhibitor was L-Cysteine requiring a lower concentration compared to ascorbic acid and fermented extract. However, it must be emphasized that both L-Cysteine and ascorbic acid are pure inhibitors that at low concentrations manage to slow down this enzymatic activity. On the other hand, the fermented extract is a mixture of different compounds that in the current research have not been identified; therefore, this may influence its concentration to generate a complete inhibition.

L-Cysteine exhibited more accelerated inhibition when used at low concentrations. Nevertheless, a similar trend was observed for the ascorbic acid and the fermented extract when they were at concentrations of 1.0 at 4.0 % and 10 at 30 %, respectively (Fig. 3.). The three inhibitors showed a similar behavior in the inhibition of PPO.

The inhibition of PPO extracted from avocado variety Booth with L-Cysteine at 0.20 mM was close to 60 % and with ascorbic acid at 0.5 mM was 80 % [4], these values are lower than the results found, since, at 2 % (0.20 mM) of L-Cysteine, and 5 % (0.5 mM) of ascorbic acid an inhibition of 69 % and 92 % was obtained, respectively, although the differences may be due to the characteristics of each variety of avocado. In the case of Boot 8 variety, it has been reported total phenols 1.2 ± 0.1 mg eq GAE/g, chlorophyll α (2.7 ± 0.7 μg/g), chlorophyll β (3.0 ± 1.1ug/g) and carotenoids (3.4 ± 0.7 μg/g). These compounds may explain the enzyme inhibition [49].

The inhibition of PPO by L-Cysteine occurs through the formation of stable complexes with the copper present in the active site of the enzyme; nonetheless, the concentrations of L-Cysteine necessary to reach acceptable levels of browning inhibition have negative effects on the sensory properties of the treated foods. In turn, ascorbic acid reduces the o-quinones to their original phenols, but once the ascorbic acid is oxidized, the o-quinones are no longer reduced and can undergo oxidative polymerization to produce dark brown melanin pigments [31].

In a study previous to this work, avocado seed extract was characterized concerning its antioxidant potential and it was obtained that ABTS (1690.634 mg TE/100 g ± 22.852) and DPPH (15946.922 mg TE/g ± 349.857), also, some polyphenolic compounds with antioxidant capacity that were identified in the extract are epicatechin (13.506 mg/g), catechin (8.825 mg/g), procyanidin (3.633) and gallic acid (1.464 mg/g) [36] The fermented extract showed that it contains potential inhibitors capable of slowing the darkening of the avocado. Compounds such as catechins, epicatechin, procyanidin and gallic acid possess antioxidant properties that help reduce the formation of reactive oxygen species (ROS) and hydrogen peroxide (H₂O₂), which are involved in the browning process. This dual action—both inhibiting the enzyme and reducing oxidative stress—contributes to the overall anti-browning effect of catechins in various food products [33].

The hydroxyl groups present in the polyphenol compounds could carry out a nucleophilic attack on the copper atoms present in the active site of the PPO and participate directly in the transfer of protons during the catalysis, with its subsequent inhibition [50,51]. Other researchers have reported similar results of the inhibitory capacity of some natural extracts, for instance, the onion extract subjected to heating could inhibit the PPO and thus delay the darkening in the pear juice [50], likewise, the addition of 30 mg/mL of green tea extract resulted in approximately 42 % inhibition of PPO in apple [12], 75 mg/L of grape seed extract inhibited the PPO of lettuce by 50 % [4], the brown rice extract generated an inhibition of 69.31 % in potato and of 47.63 % in banana [18]. Although phenolic compounds act as antioxidants as well as substrates for browning reactions, there is great interest in studying the positive influence of these compounds on the enzymatic browning reaction [52]. Some authors have reported that PPO inhibitors have specific modes of action such as reducing agents, chelating agents, complexing agents, acidulants, enzyme inhibitors, and enzyme treatments [1].

**Fig. 4:**
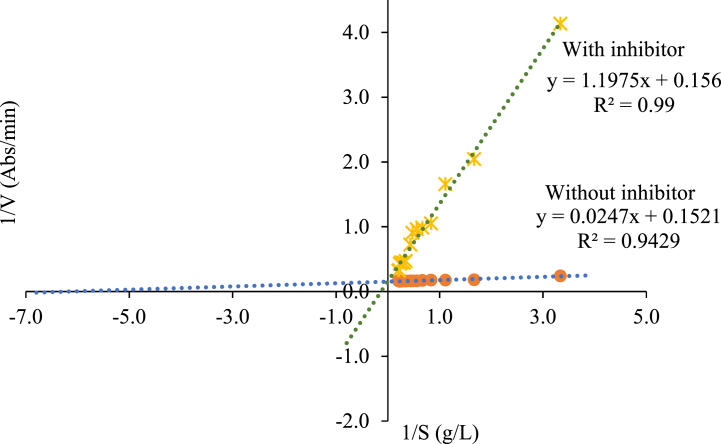
Lineweaver-Burk linearization. Inhibitory effect of Fermented Extract on the enzymatic activity of the PPO. ∗ With inhibitor, **.**Without inhibitor.

Fig. 4 presents the linearization of the reaction speed to the catechol substrate with and without addition of fermented extract; as a result, lines with different slopes and intersections are generated on the "Y" axis, which is related to the type of competitive inhibition [53]. The maximum speed remained constant, but the km increased significantly when going from 1.48 mM without an inhibitor to 49.7 mM in the presence of the fermented extract that acts as an inhibitor. This change in Km indicates a lower affinity of the PPO enzyme because of the catechol substrate. The substances that cause competitive inhibition have molecular similarity to the substrate, the inhibitor competes with the substrate for binding to the active centers of the enzyme, thus preventing part of it from forming the enzyme-substrate complex; conversely, there is formation of the complex enzyme-inhibitor that is catalytically inactive [[53], [54], [55]]. As a result, the fermented extract acts by reducing the concentration of the free PPO for the fixation of the substrate.

### 3.3 Browning index (BI)

**Fig. 5:**
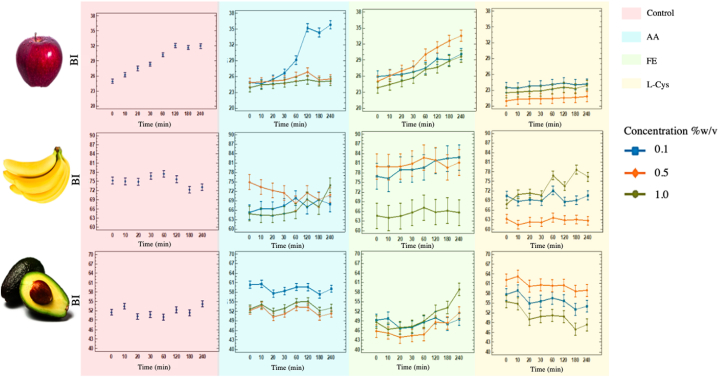
Browning Index of apple, banana, and avocado pulp treated with different inhibitors: Ascorbic acid (AA), Fermented Extract (FE), and L-Cysteine (L-Cys); at different concentrations (0.1, 0.5, and 1.0%w/v).

Fig. 5 shows the effect of AA, FE, and L-Cys on the browning index (BI) of apples, bananas, and avocado pulp at three different concentrations (0.1, 0.5, and 1.0 % w/v), compared to the control (Fruit without added inhibitor). Regarding the inhibition with AA, it was observed the concentration of 1.0 % presented the lowest BI in all times evaluated in apples, and most of the time in bananas, while in avocado pulp the lowest BI occurred at 0.5 %. The FE had the same trend as ascorbic acid, at 1.0 % the lowest BI was presented in apple and banana, while in avocado at 0.5 %. L-Cys had the lowest BI at 0.5 % in apples and bananas, whereas in avocado it was presented at 1.0 %.

The results showed that the FE treatment exhibited much lighter browning than the control which indicated that FE had a significant effect on inhibiting browning by PPO action, this effect could be attributed to the antioxidant properties of the phenolic compounds present in FE, these compounds could interact with protein or enzymes via hydrogen bond or hydrophobic interaction or by interacting with the active site of the enzyme [56]. In addition, these may act as competitive inhibitors to PPO [57]. In contrast, other authors have reported the anti-browning function of natural agents with antioxidant capacity, such as green tea extract and honey extracted from different floral sources applied in freshly cut apples [52,58,59]. Similarly, Purslane (*Portulaca oleracea*), a popular edible wild herb in China, could effectively inhibit PPO activity and browning index of freshly cut potato slices at 0.05 % aqueous extract which was also found rich in polyphenolic compounds [60]. Likewise, Tinello & Lante [61] found a high concentration of (−)-Epigallocatechin gallate in the unripe juices of two red grape cultivars (*Vitis vinifera* cvs Barbera and Merlot) that exhibited strong antioxidant and anti-browning properties and limited the color change of fresh-cut apples and potatoes. Phenolic compounds display strong antioxidant activity and therefore have the potential as oxidative enzyme inhibitors, thus, it has increased the interest in finding natural antioxidants, especially in plants [62].

The anti-browning effect of ascorbic acid and or Cysteine has also been compared with plant extracts rich in polyphenolic compounds [[63], [64], [65]]. For example, strawberry tree leaves extract and apple byproduct extract in fresh-cut pears had similar behavior to ascorbic acid by inhibiting 50 % of the enzyme activity and by retarding the browning index [62]. Also, it was found that the enzymatic browning of stored mung bean sprouts can be drastically reduced by ascorbic acid since at 20 mM the activity of PPO was reduced by 97 % [66]. Other recent work showed low-dose L-Cys treatment at the concentration of 0.01 % could prevent the browning incidence of lotus root slices [67] and, Son et al. [68] suggested 1 % L-Cys immersion inhibited browning of apple slices being greater than 1 % ascorbic immersion.

Antioxidant solutions, like ascorbic acid, and its derivatives, and sulfur-containing compounds such as cysteine, have been used for a long time to control browning in freshly cut fruits however, some could leave unpleasant sensory effects, have short-term function, high cost, or potential health impacts [52,68]. Likewise, ascorbic acid is very unstable and oxidized especially in the presence of air and light which represents a disadvantage in its use [69].

**Fig. 6:**
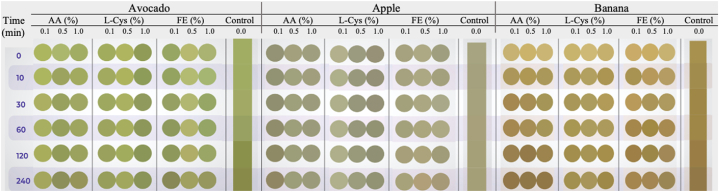
Unified vector kinetics of colorimetry for avocado pulp, apple, and banana.

Darkening or discoloration of freshly cut fruits and vegetables is a critical indicator of loss of freshness and quality. Fig. 6 shows the monitoring over time of the color change of the avocado pulp treated with AA, L-Cys, EF, and control (distilled water). This figure confirms that the avocado pulp treated with 0.5 % w/v of AA and EF exhibited lower BI and therefore less affectation to color, while L-Cys had better action at 1.0 % w/v. A fading of the original green color was noted in the other evaluated concentrations of the three inhibitors. In apple and banana, the treatments evaluated delayed the changes in color attributes rather than the control treatment, nevertheless, a greater affectation in the color over time was evidenced compared to the avocado pulp. Probably, the inhibitor AA both in apple and banana, suffered self-oxidation with the subsequent accumulation of o-quinones which is derived in colored complexes [70].

### 3.4 Mathematical modeling of the change in the browning index (BI)

**Fig. 7:**
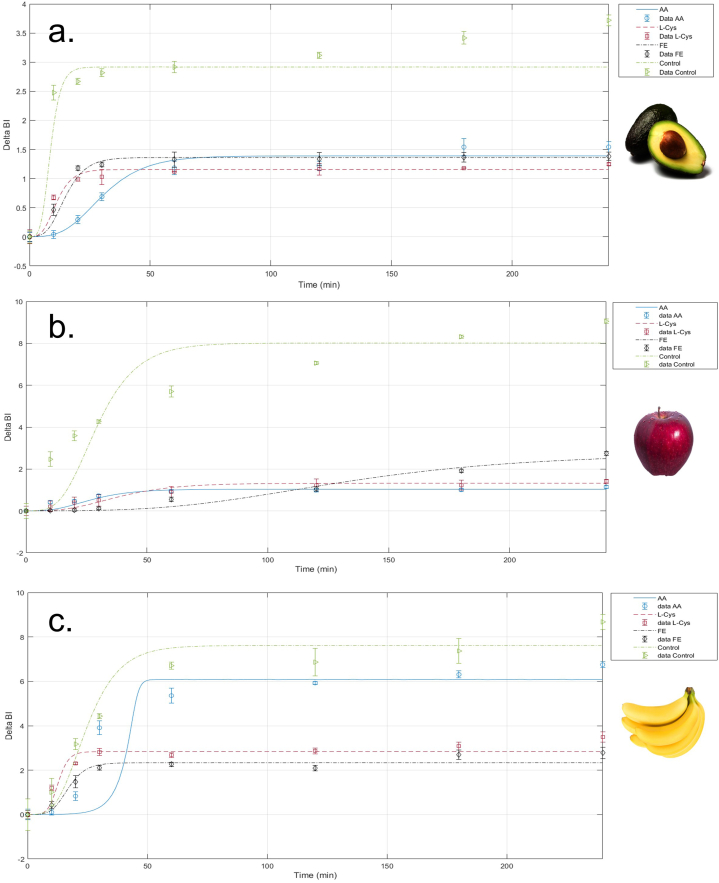
Browning index change (ΔBI) of a. Avocado pulp at 0.5 % of each inhibitor, b. Apple at 1.0 % of each inhibitor, c. Banana at 1.0 % of each inhibitor.

Fig. 7a shows the kinetics of ΔBI in avocado pulp at 0.5 %, since it was the concentration that presented less browning by the FE and by AA. After 30 min the FE and the L-Cysteine stabilized the BI, while the ascorbic acid did so after 60 min and presented an initial latency period until 10 min with a subsequent exponential increase. Whereas, the L-Cysteine showed a delay period similar to the fermented extract. This result could be explained by the effect that L-Cysteine has on reacting with the quinones formed during the initial phase of enzymatic browning to produce colorless complexes or to reduce o-quinones to o-diphenols [70]. The avocado pulp treated with EF at 0.5 % w/v exhibited lower browning indices concerning the control, and, from min 60 it presents the same behavior of ascorbic acid. This result is directly related to the inhibitory effect on the PPO, which reveals that the fermented extract is an inhibitor with similar capacity to L-Cysteine and ascorbic acid as well. The kinetics parameters are shown in Table 2. For avocado pulp, all inhibitors presented sensitivity to self-inhibition due to n parameter was less than 1.

**Table 2:** Kinetic parameters.

Inhibitors	Avocado			Apple			Banana		
	Vmax (ΔBI/min)	ΔBImax	n	Vmax (ΔBI/min)	ΔBImax	n	Vmax (ΔBI/min)	ΔBImax	n
AA	1,284	1,390	0,067	20,785	1,037	0,004	0,193	6,084	3,920
L-Cys	2,425	1,156	0,060	5,108	1,325	0,012	0,719	2,836	0,556
FE	1,817	1,362	0,100	3,135	2,731	0,006	0,702	2,334	0,320
Control	2,052	3,651	0,280	5,983	9,014	0,005	7,187	7,614	0,015

In the kinetics of ΔBI in apple at 1.0 % (Fig. 7b.), it was evidenced that EF had excellent protection against browning up to 120 min of the analysis, however, from that moment the ΔBI increased gradually. ΔBI with AA and L-Cys increased during the first 50 min and remained constant thereafter. In the same way, all inhibitors presented sensitivity to self-inhibition due to n parameter was less than 1.

In banana, EF presented lower values of ΔBI compared to L-Cys (Fig. 7c.),. While the AA had a behavior similar to the control, which indicates that it did not present good protection against browning. The n kinetic parameter for AA presented a value greater than 1, which indicates resistance to self-inhibition, the other two inhibitors presented sensitivity to self-inhibition.

In another study, a comparison between the first-order model and the Weibull model was regarding their suitability to fit the enzymatic browning kinetic data in samples of mushrooms, pears, apples, avocados, and bananas, based on the use of the L∗ luminosity, the ΔE∗, and the BI as the browning indexes, similar to our results, they found the β value was different to 1; in apples, it was 0.29, in avocados 0.53, and 0.48 in bananas, which indicates the browning kinetics of the samples is not following precisely a first order kinetic [71]. The power-law model used to model the enzymatic kinetic of fractal dimension value (FDL∗) also has been used for fruits and foods that have a non-homogenous color surface during enzymatic browning kinetics [72].

## 4 Conclusions

The present study has demonstrated the biological activity as a PPO inhibitor and the anti-browning potential of the fermented extract of the avocado seed. The Km and Vmax kinetic parameters of avocado PPO, using catechol as substrate, were 0.1627 g/L and 0.6577 Abs/min, respectively. The fermented extract completely inhibited the enzyme at 60 % v/v, and its type of inhibition was competitive. Additionally, its anti-browning effect could be evidenced in avocado (0.5%w/v), apple (1.0%w/v), and banana (1.0%w/v), since effectively retarded browning incidence which is related to the presence of phenolic compounds as potential inhibitors. The present research provides promising results on the use that can be given to the fermented extract as an agent for the prevention of browning caused by the PPO, which additionally indicates the potential use that the avocado seed has for the extraction of the bioactive compounds.

## CRediT authorship contribution statement

**Diana Paola Yepes-Betancur:** Investigation. **Isabel Cristina Zapata-Vahos:** Investigation. **Juan Camilo Henao-Rojas:** Software, Data curation. **Jaison Martinez-Saldarriaga:** Investigation. **Carlos Julio Márquez-Cardozo:** Project administration. **Edith Marleny Cadena-Chamorro:** Project administration.

## Data availability statement

Data will be made available on request.

## Declaration of competing interest

The authors declare that they have no known competing financial interests or personal relationships that could have appeared to influence the work reported in this paper.

## References

[1] Yoruk R., Marshall M.R. (2003). Physicochemical properties and function of plant polyphenol oxidase: a review. J. Food Biochem..

[2] Espín J.C., Jolivet S., Wichers H.J. (1998). Inhibition of mushroom polyphenol oxidase by agaritine. J. Agric. Food Chem..

[3] Van Gelder C.W., Flurkey W.H., Wichers H.J. (1997). Sequence and structural features of plant and fungal Tyrosinases. Phytochemistry.

[4] Altunkaya A., Gökmen V. (2012). Effect of grape leaf extract on phenolic profile and browning of fresh-cut lettuce (Lactucasativa). J. Food Biochem..

[5] Kahn V. (1985). Effect of proteins, protein hydrolyzates and amino acids on o‐dihydroxyphenolase activity of polyphenol oxidase of mushroom, avocado, and banana. J. Food Sci..

[6] Espín J.C., Trujano M.F., Tudela J., García-Cánovas F. (1997). Monophenolase activity of polyphenol oxidase from hass avocado. J. Agric. Food Chem..

[7] Weemaes C., Ludikhuyze L., Van den Broeck I., Hendrickx M. (1998). Effect of pH on pressure and thermal inactivation of avocado Polyphenol Oxidase: a kinetic study. J. Agric. Food Chem..

[8] Soliva-Fortuny R., Elez-Martínez P., Sebastián-Calderó M., Martín-Belloso O. (2002). Kinetics of polyphenol oxidase activity inhibition and browning of avocado puree preserved by combined methods. J. Food Eng..

[9] Gui F., Wu J., Chen F., Liao X., Hu X., Zhang Z., Wang Z. (2007). Inactivation of polyphenol oxidases in cloudy apple juice exposed to supercritical carbon dioxide. Food Chem..

[10] Yemenicioǧlu A., Özkan M., Cemeroǧlu B. (1997). Heat inactivation kinetics of apple Polyphenoloxidase and activation of its latent form. J. Food Sci..

[11] Manzocco L., Plazzotta S., Spilimbergo S., Nicoli M.C. (2017). Impact of high-pressure carbon dioxide on polyphenoloxidase activity and stability of fresh apple juice. LWT - Food Sci. Technol. (Lebensmittel-Wissenschaft -Technol.).

[12] Moreno J., Simpson R., Pizarro N., Pavez C., Dorvil F., Petzold G., Bugueño G. (2013). Influence of ohmic heating/osmotic dehydration treatments on Polyphenoloxidase inactivation, physical properties and microbial stability of apples (cv. Granny Smith). Innov. Food Sci. Emerg. Technol..

[13] Siddiq M., Sinha N.K., Cash J.N. (1992). Characterization of Polyphenoloxidase from stanley plums. J. Food Sci..

[14] Siddiq M., Arnold J., Sinha N., Cash J. (1994). Effect of Polyphenol Oxidase and its inhibitors on anthocyanin changes in plum juice. J. Food Process. Preserv..

[15] Montgomery M.W., Sgarbieri V.C. (1975). Isoenzymes of banana polyphenol oxidase. Phytochemistry.

[16] Yang C.-P., Fujita S., Ashrafuzzaman M., Nakamura N., Hayashi N. (2000). Purification and characterization of Polyphenol Oxidase from banana (Musa sapientum L.) pulp. J. Agric. Food Chem..

[17] Landi M., Degl'Innocenti E., Guglielminetti L., Guidi L. (2013). Role of ascorbic acid in the inhibition of polyphenol oxidase and the prevention of browning in different browning-sensitive Lactuca sativa var. capitata (L.) and Eruca sativa (Mill.) stored as fresh-cut produce. J. Sci. Food Agric..

[18] Sukhonthara S., Kaewka K., Theerakulkait C. (2016). Inhibitory effect of rice bran extracts and its phenolic compounds on polyphenol oxidase activity and browning in potato and apple puree. Food Chem..

[19] Zhang L., Dai S., Brannan R. (2017). Effect of high pressure processing, browning treatments, and refrigerated storage on sensory analysis, color, and Polyphenol Oxidase activity in pawpaw (Asimina triloba L.) pulp. LWT - Food Sci. Technol. (Lebensmittel-Wissenschaft -Technol.).

[20] Fang C., Wang C., Xiong Y.L., Pomper K.W. (2007). Extraction and characterization of polyphenol oxidase in pawpaw (Asimina Triloba) fruit. J. Food Biochem..

[21] Gouzi H., Depagne C., Coradin T. (2012). Kinetics and thermodynamics of the thermal inactivation of polyphenol oxidase in an aqueous extract from *Agaricus bisporus*. J. Agric. Food Chem..

[22] Yi J., Jiang B., Zhang Z., Liao X., Zhang Y., Hu X. (2012). Effect of ultrahigh hydrostatic pressure on the activity and structure of mushroom (*Agaricus bisporus)* polyphenoloxidase. J. Agric. Food Chem..

[23] Zhou L., Xiong Z., Liu W., Zou L. (2017). Different inhibition mechanisms of gentisic acid and cyaniding-3-O-glucoside on polyphenoloxidase. Food Chem..

[24] Nirmal N.P., Benjakul S. (2009). Effect of ferulic acid on inhibition of polyphenoloxidase and quality changes of Pacific white shrimp (Litopenaeus vannamei) during iced storage. Food Chem..

[25] Zhou L., Liu W., Xiong Z., Zou L., Chen J., Liu J., Zhong J. (2016). Different modes of inhibition for organic acids on polyphenoloxidase. Food Chem..

[26] Kahn V. (1976). Effect of some phenolic compounds on the oxidation of 4-methil catechol catalyzed by avocado polyphenoloxidase. J. Food Sci..

[27] Kim M.J., Young C., Park I. (2005). Prevention of enzymatic browning of pear by onion extract. Food Chem..

[28] Soysal Ç. (2009). Effects of green tea extract on “Golden delicious” apple Polyphenoloxidase and its browning. J. Food Biochem..

[29] Altunkaya A., Gökmen V. (2012). Effect of grape leaf extract on phenolic profile and browning of fresh-cut lettuce (Lactucasativa). J. Food Biochem..

[30] Duan X., Su X., Shi J., You Y., Zhao M., Li Y., Wang Y., Jiang Y. (2008). Inhibitory effect of anthocyanin extract from seed coat of black bean on pericarp browning and lipid peroxidation of litchi fruit during storage. J. Food Biochem..

[31] Walker J.R.L., Lee C.Y., Whitaker J.R. (1995). Enzymatic Browning and its Prevention.

[32] Kupnik K., Primožič M., Kokol V., Knez Ž., Leitgeb M. (2023). Enzymatic, antioxidant, and antimicrobial activities of bioactive compounds from avocado (Persea americana L.) seeds. Plants.

[33] Shevkani K. (2023). Food-based natural mitigators of enzymatic browning on fruits and vegetables: insights into active constituents. Modes of Action, and Challenges, Food Bioproc Tech.

[34] Márquez C., Yepes D., Sanchez L. (2014). Cambios físicos-químicos del aguacate (Persea americana Mill. cv. “Hass”) en poscosecha para dos municipios de Antioquia. Temas Agrarios.

[35] Fang C., Wang C., Xiong Y.L., Pomper K.W. (2007). Extraction and characterization and of polyphenol oxidase in pawpaw (Asimina triloba) fruit. J. Food Biochem..

[36] Yepes-Betancur D.P., Márquez-Cardozo C.J., Cadena-Chamorro E.M., Martinez-Saldarriaga J., Torres-León C., Ascacio-Valdes A., Aguilar C.N. (2021). Solid-state fermentation – assisted extraction of bioactive compounds from hass avocado seeds. Food Bioprod. Process..

[37] Sukhonthara S., Theerakulkait C. (2012). Inhibitory effect of rice bran extract on polyphenol oxidase of potato and banana. Int. J. Food Sci. Technol..

[38] Palou E., López‐Malo A., Barbosa‐Cánovas G.V., Welti‐Chanes J., Swanson B.G. (1999). Polyphenoloxidase activity and color of blanched and high hydrostatic pressure treated banana puree. J. Food Sci..

[39] Montoya S., Sánchez Óscar J., Levin Laura (2015). Production of lignocellulolytic enzymes from three white-rot fungi by solid-state fermentation and mathematical modeling. Afr. J. Biotechnol..

[40] Henao-Rojas J., Rodríguez P. (2016). Evaluation of color during avocado (Persea americana Mill. cv. Hass) ripening, Agron Colomb. Agron. Colomb..

[41] Butcher J. (1987).

[42] Gómez F., Sánchez S., Iradi M., Azman N., Almajano M. (2014). Avocado seeds: extraction optimization and possible use as antioxidant in food. Antioxidants.

[43] Sayavedra‐Soto L.A., Montgomery M.W. (1986). Inhibition of polyphenoloxidase by sulfite. J. Food Sci..

[44] McLarin M.-A., Leung I.K.H. (2020). Substrate specificity of polyphenol oxidase. Crit. Rev. Biochem. Mol. Biol..

[45] Tilley A., McHenry M.P., McHenry J.A., Solah V., Bayliss K. (2023). Enzymatic browning: the role of substrates in polyphenol oxidase mediated browning: mechanisms of enzymatic browning. Curr. Res. Food Sci..

[46] Amaya E., Tarkus R., Dominguez M. (2008). Extracción y caracterización cinética de la enzima Polifenoloxidasa del aguacate (Persea americana MILLER) Var. Hass, Revista de La Facultad de Ingeniería Química. Universidad Autónoma de Yucatán.

[47] Gómez-López V.M. (2002). Some biochemical properties of polyphenol oxidase from two varieties of avocado. Food Chem..

[48] Broothaerts W., McPherson J., Li B., Randall E., Lane W.D., Wiersma P.A. (2000). Fast apple (malus domestica) and tobacco (nicotiana tobacum) leaf polyphenol oxidase activity assay for screening transgenic plants. J. Agric. Food Chem..

[49] Wang W., Bostic T.R., Gu L. (2010). Antioxidant capacities, procyanidins and pigments in avocados of different strains and cultivars. Food Chem..

[50] Kim M.-J., Kim C.Y., Park I. (2005). Prevention of enzymatic browning of pear by onion extract. Food Chem..

[51] Xiong Z., Liu W., Zhou L., Zou L., Chen J. (2016). Mushroom (Agaricus bisporus) polyphenoloxidase inhibited by apigenin: multi-spectroscopic analyses and computational docking simulation. Food Chem..

[52] Jeon M., Zhao Y. (2005). Honey in combination with vacuum impregnation to prevent enzymatic browning of fresh-cut apples. Int. J. Food Sci. Nutr..

[53] Voet D., Voet J.G., Pratt C.W. (2007). Fundamentals of biochemestry: life at the molecular level.

[54] Berg J., Tymoczko J., Stryer L. (2007).

[55] Doran P.M. (2012).

[56] Le Bourvellec C., Renard C.M.G.C. (2012). Interactions between polyphenols and macromolecules: quantification methods and mechanisms. Crit. Rev. Food Sci. Nutr..

[57] Tinello F., Lante A. (2018). Recent advances in controlling polyphenol oxidase activity of fruit and vegetable products. Innov. Food Sci. Emerg. Technol..

[58] Soysal Ç. (2009). Effects of green tea extract on “Golden delicious” apple Polyphenoloxidase and its browning. J. Food Biochem..

[59] Moreno J., Simpson R., Pizarro N., Pavez C., Dorvil F., Petzold G., Bugueño G. (2013). Influence of ohmic heating/osmotic dehydration treatments on Polyphenoloxidase inactivation, physical properties and microbial stability of apples (cv. Granny Smith). Innov. Food Sci. Emerg. Technol..

[60] Liu X., Yang Q., Lu Y., Li Y., Li T., Zhou B., Qiao L. (2019). Effect of purslane (Portulaca oleracea L.) extract on anti-browning of fresh-cut potato slices during storage. Food Chem..

[61] Tinello F., Lante A. (2017). Evaluation of antibrowning and antioxidant activities in unripe grapes recovered during bunch thinning. Aust. J. Grape Wine Res..

[62] Dias C., Fonseca A.M.A., Amaro A.L., Vilas-Boas A.A., Oliveira A., Santos S.A.O., Silvestre A.J.D., Rocha S.M., Isidoro N., Pintado M. (2020). Natural-based antioxidant extracts as potential mitigators of fruit browning. Antioxidants.

[63] Yu K., Zhou L., Sun Y., Zeng Z., Chen H., Liu J., Zou L., Liu W. (2021). Anti-browning effect of Rosa roxburghii on apple juice and identification of polyphenol oxidase inhibitors. Food Chem..

[64] Zeng F., Ge Z., Limwachiranon J., Li L., Feng S., Wang Y., Luo Z. (2017). Antioxidant and tyrosinase inhibitory activity of *Rosa roxburghii* fruit and identification of main bioactive phytochemicals by <scp>UPLC</scp> ‐Triple‐ <scp>TOF</scp>/<scp>MS</scp&gt. Int. J. Food Sci. Technol..

[65] Awad M.A., Al-Qurashi A.D., Alrashdi A.M.A., Mohamed S.A., Faidi F. (2017). Developmental changes in phenolic compounds, antioxidant capacity and enzymes activity in skin of ‘El-Bayadi’ table grapes. Sci. Hortic..

[66] Sikora M., Świeca M. (2018). Effect of ascorbic acid postharvest treatment on enzymatic browning, phenolics and antioxidant capacity of stored mung bean sprouts. Food Chem..

[67] Wen B., Li D., Tang D., Huang Z., Kedbanglai P., Ge Z., Du X., Supapvanich S. (2020). Effects of simultaneous ultrasonic and cysteine treatment on antibrowning and physicochemical quality of fresh-cut lotus roots during cold storage. Postharvest Biol. Technol..

[68] Son S.M., Moon K.D., Lee C.Y. (2001). Inhibitory effects of various antibrowning agents on apple slices. Food Chem..

[69] Fattahifar E., Barzegar M., Ahmadi Gavlighi H., Sahari M.A. (2018). Evaluation of the inhibitory effect of pistachio (Pistacia vera L.) green hull aqueous extract on mushroom tyrosinase activity and its application as a button mushroom postharvest anti-browning agent. Postharvest Biol. Technol..

[70] Rojas-Graü M.A., Soliva-Fortuny R., Martín-Belloso O. (2009). Edible coatings to incorporate active ingredients to fresh-cut fruits: a review. Trends Food Sci. Technol..

[71] Quevedo R., Díaz O., Valencia E., Pedreschi F., Bastias J.M., Siche R. (2016). Differences between the order model and the Weibull model in the modeling of the enzymatic browning. Food Bioprocess Technol..

[72] Noshad M., Mohebbi M., Ansarifar E., behbahani B.A. (2015). Quantification of enzymatic browning kinetics of quince preserved by edible coating using the fractal texture Fourier image. J. Food Meas. Char..

